# Hospital surveillance of respiratory viruses during the COVID-19 pandemic and beyond: contribution to the WHO mosaic framework, Israel, 2020 to 2023

**DOI:** 10.2807/1560-7917.ES.2024.29.32.2300634

**Published:** 2024-08-08

**Authors:** Lea Gur-Arie, Michal Stein, Hanna Sefty, Ilana S Fratty, Ital Nemet, Limor Kliker, Nofar Atari, Neta S Zuckerman, Alina Rosenberg, Heftziba Ivgi, Orit Golan-Shany, Nadav Sorek, Orna Schwartz-Harari, Michal Bromberg, Lital Keinan-Boker, Michal Mandelboim, Aharona Glatman-Freedman, on behalf of the Israeli Respiratory Viruses Hospital Laboratory Network (IRVHLN)

**Affiliations:** 1 Israel Center for Disease Control, Israel Ministry of Health, Tel Hashomer, Ramat Gan, Israel; 2 Pediatric Infectious Diseases Unit, Safra Children’s Hospital, Sheba Medical Center, Tel Hashomer, Ramat Gan, Israel; 3 Faculty of Medical and Health Sciences, Tel Aviv University, Tel Aviv, Israel; 4 Central Virology Laboratory, Ministry of Health, Tel Hashomer, Ramat Gan, Israel; 5 Immunology and Virology Laboratory, Sha'are-Zedek Medical Center, Jerusalem, Israel; 6 Bnai-Zion Medical Center, Microbiology Laboratory, Haifa, Israel; 7 Microbiology Laboratory, Assuta Ashdod University Hospital, Ashdod, Israel; 8 Microbiology Laboratory, Edith Wolfson Medical Center, Holon, Israel; 9 School of Public Health, Faculty of Medical and Health Sciences, Tel Aviv University, Tel Aviv, Israel; 10 School of Public Health, University of Haifa, Haifa, Israel; 11 The members of the Israeli Respiratory Viruses Hospital Laboratory Network (IRVHLN) are acknowledged at the end of the article

**Keywords:** respiratory viruses, surveillance, mosaic framework, influenza virus, respiratory syncytial virus, RSV, surveillance, COVID-19, pandemic, SARS-CoV-2

## Abstract

**Background:**

A new respiratory virus surveillance platform, based on nationwide hospital laboratory data, was established in Israel during the COVID-19 pandemic.

**Aim:**

We aimed to evaluate the performance of this platform with respect to the detection of influenza and respiratory syncytial virus (RSV) from week 36 in 2020 to week 15 in 2023, and how it fits with the World Health Organization (WHO) mosaic surveillance framework.

**Methods:**

Data of respiratory samples from hospitalised patients sent for laboratory confirmation of influenza virus or RSV from 25 general hospital laboratories nationwide were collected. We analysed the weekly number and percentage of samples positive for influenza virus or RSV vis-à-vis SARS-CoV-2 activity and compared data from the new surveillance platform with existing surveillance platforms. Using data in the new surveillance platform, we analysed early stages of a 2021 out-of-season RSV outbreak and evaluated the capabilities of the new surveillance system with respect to objectives and domains of the WHO mosaic framework.

**Results:**

The new hospital-laboratory surveillance platform captured the activity of influenza virus and RSV, provided crucial data when outpatient sentinel surveillance was not operational and supported an out-of-season RSV outbreak investigation. The new surveillance platform fulfilled important objectives in all three domains of the mosaic framework and could serve for gathering additional information to fulfil more domain objectives.

**Conclusion:**

The new hospital laboratory surveillance platform provided essential data during the COVID-19 pandemic and beyond, fulfilled important domain objectives of the mosaic framework and could be adapted for the surveillance of other viruses.

**Key public health message**

**What did you want to address in this study and why?**

The COVID-19 pandemic highlighted the need to broaden and strengthen respiratory virus surveillance. During the pandemic, the Israel Center for Disease Control created a new platform for surveillance of respiratory viruses. We wanted to evaluate the performance of this new platform and compare it with existing surveillance and the WHO recommendations for a respiratory virus surveillance framework.

**What have we learnt from this study?**

The new surveillance platform could reliably capture the activity of influenza virus and respiratory syncytial virus (RSV) during the COVID-19 pandemic and beyond. It filled crucial gaps of other surveillance tools during the pandemic years and detected an out-of-season RSV outbreak. The new surveillance platform can also be adapted to the surveillance of other viruses.

**What are the implications of your findings for public health?**

It is important to continuously evaluate the capabilities of surveillance platforms and their ability to adapt to changing circumstances, such as pandemics. Furthermore, our study demonstrates the usefulness of the WHO respiratory virus mosaic framework for assessing the capabilities of new surveillance platforms.

## Introduction

As part of a preparation for a post COVID-19 pandemic period, as well as for future pandemics, the World Health Organization (WHO) mosaic surveillance framework was proposed [1]. This proposal encourages countries to increase the number of effective surveillance approaches for monitoring the activity of respiratory viruses that can potentially cause epidemics and/or pandemics [1]. The different approaches should complement one another to provide a comprehensive representation of various elements of outbreaks of respiratory viruses and meet various surveillance objectives [1]. These surveillance objectives can be grouped into three surveillance domains: detection and assessment, monitoring epidemiological characteristics and informing the use of human health intervention.

Israel’s respiratory virus surveillance system, conducted by the Israel Center for Disease Control (ICDC), has traditionally relied on the use of several surveillance platforms. These included primary care syndromic surveillance, based on data from two of the four health maintenance organisations (HMO) in Israel which cover ca 80% of the population, emergency department (ED) syndromic surveillance, laboratory-based primary care sentinel surveillance and laboratory-based severe acute respiratory infection (SARI) hospital surveillance [2,3]. While syndromic surveillance data are being received at the ICDC regularly throughout the year, the laboratory-based surveillance platforms (primary care sentinel and SARI) collected data usually during the autumn and winter months. During the COVID-19 pandemic primary care sentinel clinics collected data also during other periods of the year.

The COVID-19 pandemic presented notable challenges to the surveillance of respiratory viruses in Israel. A national repository for test results of severe acute respiratory syndrome coronavirus 2 (SARS-CoV-2) was established in the early stages of the pandemic, automatically capturing all official SARS-CoV-2 tests performed [4]. While this repository is still operational, similar repositories do not exist for other respiratory viruses.

During the early phases of the COVID-19 pandemic, visits due to acute infectious respiratory syndromes declined in both primary care clinics and EDs [3]. This decrease was partly due to the guidelines of the Ministry of Health (such as those pertaining to quarantine and physical visits to medical facilities by symptomatic individuals) and partly due to public concern of exposure to SARS-CoV-2-positive individuals [3]. In addition, there was no correlation between the pattern of upper respiratory infection (URI) and SARS-CoV-2 activity [3]. Thus, the syndromic surveillance platforms became unreliable.

During the COVID-19 pandemic, we were also unable to rely on the SARI surveillance platform, as the patient population in hospital departments originally assigned for SARI surveillance changed dramatically, because hospital departments were assigned to care for either SARS-CoV-2-positive patients or for SARS-CoV-2-negative patients.

As the syndromic surveillance and SARI surveillance platforms could not be relied upon, we were left with the primary care sentinel surveillance platform only. The primary care sentinel surveillance was adapted to SARS-CoV-2 surveillance, which started in week 13 2020 [5]; the 2020 samples collected until week 39 were tested for SARS-CoV-2 only. From week 40 2020 onwards, samples were tested also for influenza virus and respiratory syncytial virus (RSV).

Due to the concern that the circulation of respiratory viruses would be affected by the COVID-19 pandemic and that the primary care surveillance platform may not be sensitive enough to capture low viral circulation, we established a new surveillance platform relying on respiratory virus detection among hospitalised patients throughout Israel. The new platform became operational in week 36 2020. In contrast to SARI surveillance, which was conducted in selected departments before the COVID-19 pandemic and relied on the use of a case definition, this hospital surveillance platform included all general hospitals in Israel and used test results of samples which were sent by the medical teams based on clinical considerations and not a specific case definition.

In this study, we aimed to evaluate the performance of the respiratory virus hospital surveillance platform in Israel with respect to the detection of influenza virus and RSV from week 36 2020 to week 15 2023, and how it fits with the WHO mosaic surveillance framework.

## Methods

### Reporting by general hospitals

The influenza and RSV monitoring platform for hospitalised patients relies on data from all 25 general hospital laboratories nationwide (which serve 26 general hospitals), covering all Israeli residents (an average of 9.5 million during the study period). It was established as a passive surveillance platform of respiratory samples sent for laboratory confirmation based on decisions made by hospital medical teams.

Hospital laboratories reported weekly to the Israel Center for Disease Control (ICDC) the number of respiratory samples from hospitalised patients tested for influenza virus and/or RSV and the test results for these viruses. All the participating hospital laboratories used real-time (RT)-PCR for detection of influenza virus and RSV. The weekly laboratory data were sent via virtual safes established between the reporting hospitals and the ICDC to assure enhanced data protection. Reporting of findings of Influenza virus and RSV was case-based and included full name, personal identification number of the patient, test date, hospital department or unit and hospitalisation number (individually assigned number that follows the patient throughout the hospital admission).

### Hospital data analysis

Only patients admitted to the hospitals were included. If more than one sample positive for the same virus was notified from a patient within 1 month, only the first positive result was included in the analysis.

Data were aggregated by epidemiological week and presented both as raw numbers and as percentage of positive samples.

To estimate the number of deaths of hospitalised patients positive for influenza virus and RSV, we cross-referenced patient identifiers with Israel’s national population registry to identify deaths that occurred up to 30 days after sampling date.

### Comparison with other surveillance platforms

The hospital surveillance results for influenza virus and RSV were compared with the results of the laboratory-based primary care sentinel surveillance platform and the syndromic surveillance platforms of primary care and the ED. The methods of the syndromic surveillance platforms have been previously published [3,6]. Furthermore, the number of hospitalised patients positive for influenza virus or RSV per week was plotted vis-à-vis the number of hospitalised patients positive for SARS-CoV-2 per week. The latter was extracted from the national repository of SARS-CoV-2 test results, which captures hospitalisation status and hospitalisation dates of SARS-CoV-2-positive hospitalised patients (but not for SARS-CoV-2-negative hospitalised patients).

### Additional viral characterisation

Typing of influenza virus was carried out by laboratories of all general hospitals. Subtyping of influenza A virus was conducted and reported by the laboratories of two general medical centres in Israel. Since subtyping of influenza A virus is not necessary for the patient treatment, most hospital laboratories in Israel do not perform the assay. We analysed the results and compared them with the subtypes identified from primary care sentinel samples.

Typing of RSV was not carried out routinely for hospital samples or for primary care sentinel samples.

### Outbreak analysis

To demonstrate the potential of the new surveillance platform to serve as basis for outbreak investigations, we analysed the first 12 weeks of the 2021 out-of-season RSV outbreak which started in Israel in week 19 2021. We performed population analysis and genomic analysis.

#### Population analysis

To determine the population characteristics of the outbreak RSV cases, RSV-positive patients were analysed by age group, socioeconomic status (SES), and the number of household members (HHM). The analysis was performed for the patients by week of outbreak. Age was calculated based on date of birth and date of swab of the patient.

The addresses of the patients were retrieved from the population registries, and we used it to allocate each case with a geostatistical area (GSA). Israel is divided into statistical geographical areas (SGAs) based on residence address. The SGA is a small geographic unit, which usually consists of 3,000–5,000 residents who share a relatively similar economic status, living conditions, and some demographic characteristics. For each patient, the allocated GSA was used to assign the socioeconomic status (SES) cluster and the average number of members per household. The SES is determined according to the Israeli Central Bureau of Statistics (CBS) methodology. Each SGA gets an SES cluster grade from 1 (lowest) to 10 (highest), based on parameters from the Israel national census data collection, comprising household income, educational qualifications, crowding and material conditions [7]. The analysis was carried out using ArcGIS software version 10.8 (Environmental System Research Institute, Redlands, the United States (US)) with POINTS geographical layers (POINT location Intelligence, Ramat Gan, Israel). For visualisation, SES clusters were divided into categories: very low (clusters 1–4), low (clusters 5–6), medium (clusters 7–8) and high (clusters 9–10).

Statistical differences in SES for weeks 19–30, were explored using Independent-Samples Kruskal-Wallis test followed by Pairwise Comparisons between weeks adjusted using the Bonferroni correction for multiple tests. Statistical differences in number of HHM were explored using One-Way ANOVA followed by Tukey HSD multiple comparisons analysis. Statistical analysis was done using IBM SPSS Statistics for Windows, version 27 (IBM Corp. Armark, US).

Geocoding and population analyses were performed repetitively during the early stages of outbreak.

#### Genomic analysis

Early outbreak samples from five hospitals, located in different geographic areas in Israel, were analysed using whole genome next generation sequencing (NGS). Samples for sequencing were chosen as follows: RSV-positive samples received from five medical centers in Israel in the early stages of the outbreak with a quantification cycle (Cq) ≤ 30 were analysed (n = 53). Samples were screened and sequenced using Illumina RNA Prep with Enrichment (L) Tagmentation and Respiratory Virus Oligos Panel V2 kits (Illumina, San Diego, US) according to the manufacturer’s instructions. Library validation and mean fragment size was determined by TapeStation 4200 via DNA HS D1000 kit (Agilent Technologies, Santa Clara, US). Libraries were pooled, denatured, and diluted to 1 nM and sequenced on NovaSeq using the SP kit 150X2 cycles (Illumina). Sequences were mapped to RSV reference genomes (AY353550) with Burrows-Wheeler aligner (BWA) mem [8]. Multiple alignment of sequences with reference sequences was performed with multiple sequence alignment programme (MAFFT) and phylogenetic trees construction was done using Nextstrain’s Augur pipeline and visualised with Auspice [9]. To determine RSV-B genotype, only the glycoprotein (G) was used, as some whole genome sequences representing RSV-B genotypes were not available. Furthermore, the G protein gene includes approximately 3-to-4-fold higher substitution rate compared to other RSV genes [10]. To represent phylogenetic relationships between the samples analysed, whole RSV genomes were used, to provide higher resolution in terms of genomic differences.

### Contribution of the new surveillance platform to domains/objectives of the mosaic framework

The data obtained using the new surveillance platform were evaluated vis-à-vis the three domains of the mosaic framework and their 14 respective objectives [1,11].

## Results

### Influenza virus hospital surveillance

**Figure 1:**
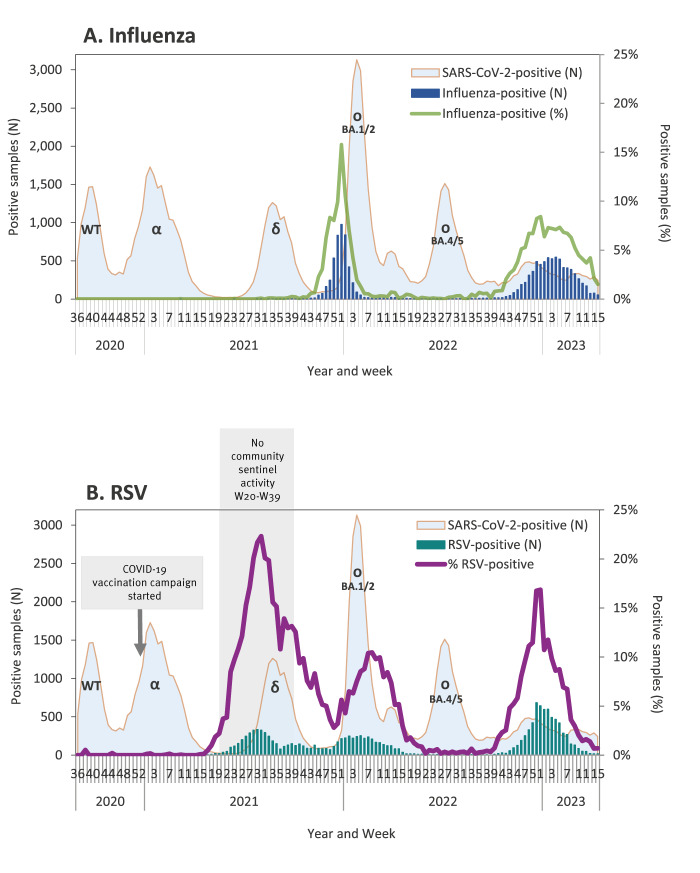
Samples from hospitalised patients positive for influenza virus and respiratory syncytial virus presented vis-à-vis hospitalised patients positive for SARS-CoV-2, by week, Israel, 2020–2023 (influenza-positive n = 12,099; RSV-positive n = 14,520; SARS-CoV-2-positive n = 83,694)

COVID-19: coronavirus disease 2019; RSV: respiratory syncytial virus; SARS-CoV-2: severe acute respiratory syndrome coronavirus 2; WT: wild-type; Greek letters denote SARS-CoV-2 variants of concern.

Figure 1A demonstrates the number of hospitalised patients positive for influenza virus vis-à-vis the number of SARS-CoV-2-positive hospital patients, by week.

During the evaluation period, we observed changes in the usual seasonal pattern of influenza virus activity. Thus, we analysed our findings by years rather than by seasons.

#### 2020–21

Between week 36 2020, starting on 5 September 2020, and week 11 2021, ending on 20 March 2021, i.e. weeks that cover the autumn and winter seasons, influenza A virus was found from one patient in week 10 2021 (Figure 1A). The epidemiological investigation of this patient revealed that influenza-like symptoms started abroad, and the patient was admitted to the hospital 1 day after arriving in Israel. Since the patient was obligated to quarantine for 10–14 days according to Israel's COVID-19 regulations for individuals coming from foreign countries, the risk for local transmission was low.

Epidemiological investigation of three other hospitalised patients tested positive for influenza virus during the same period, concluded that their illness was not caused by the notified influenza B virus, and they were excluded from the surveillance chart. One of these patients received the live-attenuated influenza vaccine (LAIV) 4 days before sampling and the clinical presentation was inconsistent with influenza. The second patient, whose positive test result was doubted by the hospital team, was positive for parainfluenza virus upon re-testing of the original sample by the Central Virology Laboratory of the Ministry of Health (MOH). The sample of the third patient had Cq 40 for the influenza virus, and the clinical presentation was inconsistent with influenza.

#### 2021–22

Low grade influenza virus activity was observed between weeks 30 and 43 2021 with up to four cases of hospitalised patients per week. The number of cases started to increase consistently in week 44 2021 reaching a peak in week 52 2021 when 976 (15.8%) of 6,179 samples tested positive for influenza virus. A consistent decline followed, paralleling the rise of cases infected with the SARS-CoV-2 Omicron variant of concern (Figure 1A). Of the 4,636 patients who tested positive for influenza virus between week 40 2021 and week 15 2022, 4,456 patients were identified in the national population registry, and 215 (4.8%) of them died within 30 days of sampling.

#### 2022–23

The nadir in hospitalised patients tested positive for influenza virus occurred between week 19 and 29 2022, with a maximum of four cases of hospitalised patients per week. A slight increase in activity occurred between weeks 30 and 39, with up to 11 cases per week. A consistent increase in the number of weekly cases was observed starting in week 40 2022, with the highest number of weekly cases between week 51 2022 and week 5 2023, and the highest percentage of positive cases detected in week 52 2022, when 451 (8.4%) of 5,357 samples were positive for influenza virus (Figure 1A). Of the 7,348 patients who tested positive for influenza virus between week 40 2022 and week 15 2023, 6,962 patients were identified in the national population registry, and 272 (3.9%) of them died within 30 days of sampling.

### Respiratory syncytial virus hospital surveillance

During the evaluation period, we observed changes in the usual seasonal pattern of RSV activity. Thus, we analysed our findings by years rather than by seasons. Figure 1B demonstrates the number of the RSV-positive hospital patients vis-à-vis the number of SARS-CoV-2-positive hospital patients, by week.

#### 2020–21

Between week 36 2020 and week 16 2021, only few sporadic RSV-positive samples were identified among hospitalised patients. Starting in week 17 2021, a consistent increase in the number of weekly cases was observed. In week 19 2021, 20 RSV-positive patients were identified, signalling out-of-season RSV circulation. The highest number of weekly RSV-positive cases was observed between week 30 and 31 2021, with the highest percentage of positive cases detected in week 31 2021 when 325 (22.3%) of 1,457 samples were RSV-positive (Figure 1B). A decline in the number and percentage of cases followed thereafter (Figure 1B). Of the 3,556 RSV-positive patients, identified between week 19 2021 and week 39 2022, 3,106 were identified in the national population registry, and 30 (1.0%) of them died within 30 days of sampling.

#### 2021–22

Following the decline in the number of RSV cases, which occurred between week 32 and 36, 2021, RSV-positive cases were consistently identified for the remainder of 2021 and into the first part of 2022. A new increase in the number and percentage of RSV-positive cases was observed starting in week 51 2021 and continued through the first weeks of 2022. This renewed activity peaked in week 8 when 210 (10.5%) of 2,008 samples were RSV-positive and subsided by week 22 2022 (Figure 1B). Of the 4,352 RSV-positive patients, identified between week 40 2021 and week 21 2022, 3,877 patients were identified in the national population registry, and 71 (1.8%) of them died within 30 days of sampling.

#### 2022–23

The nadir in RSV-positive hospitalised patients occurred between week 22 and 39 2022, with up to seven hospitalised cases per week. A consistent increase in the number of weekly cases was observed starting in week 41 2022, with the highest number of RSV-positive cases detected between week 51 and 52 2022, and the highest percentage of positive cases detected in week 52, when 642 (16.8%) of 3,811 samples were RSV-positive. By week 15 2023, the RSV activity subsided (Figure 1B). Of the 6,520 RSV-positive patients identified between week 40 2022 and week 15 2023, 5,914 patients were identified in the national population registry, and 204 (3.4%) of them died within 30 days of sampling.

### Comparison with other surveillance platforms

#### Comparison with primary care laboratory-based sentinel surveillance platform

During the 2020–21 autumn-winter months, the hospital laboratory surveillance system identified one influenza virus-positive patient while none were identified by the primary-care sentinel surveillance platform.

**Figure 2:**
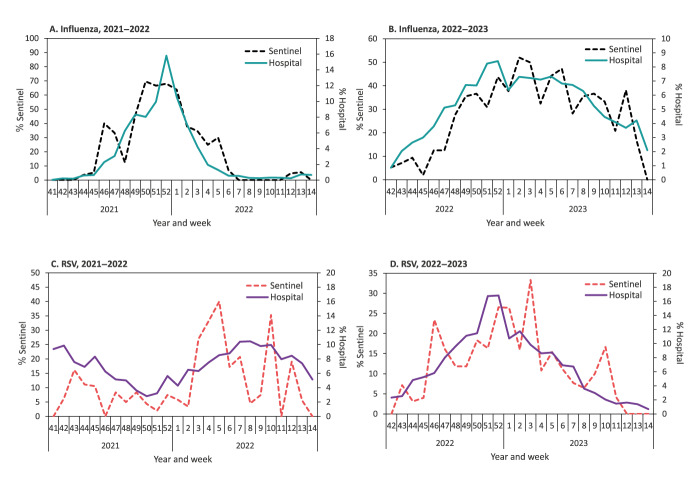
Percentage of samples from patients tested positive for influenza virus or respiratory syncytial virus: hospitalised vis-à-vis sentinel clinic patients, by week, Israel, 2021–2023

During the 2021–22 and 2022–23 seasons, the percentage of tests positive for influenza virus was substantially lower in the hospital surveillance system compared with the primary care sentinel surveillance system. However, the seasonal pattern was similar in both systems (Figures 2A and 2B).

RSV: respiratory syncytial virus.

During the 2020–21 season, the hospital surveillance platform identified only sporadic RSV-positive patients and none were detected in the primary care sentinel surveillance platform.

During the 2021–22 and 2022–23 seasons, the percentage of RSV-positive patients was substantially lower in the hospital surveillance system compared with the primary care surveillance system. However, the seasonal pattern was similar in both (Figures 2C and 2D).

#### Comparison with syndromic surveillance platforms (2020–2023)

**Figure 3:**
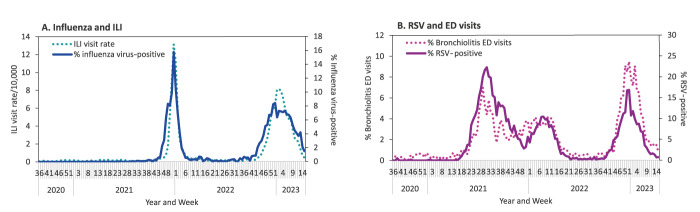
Percentage of samples from hospitalised patients tested positive for influenza virus or respiratory syncytial virus, vis-à-vis primary care visits due to influenza-like illness, or emergency departments visits due to bronchiolitis, by week, Israel, 2020–2023 (influenza-positive n = 12,099; RSV-positive n = 14,520)

ED: emergency department; ILI: influenza-like illness; RSV: respiratory syncytial virus.

The patterns of the percentage of hospital patients tested positive for influenza virus and the rate of primary care physician visits due to influenza-like illness (ILI) were similar (Figure 3A), as were the patterns of the percentage of RSV-positive hospital patients and the rate of emergency department visits due to bronchiolitis among children aged < 2 years (Figure 3B).

Panel A: Presentation of hospitalised patients tested positive for influenza virus, vis-à-vis rates of visits to primary care per 10,000 population due to influenza-like illness.

Panel B: Presentation of hospitalised patients tested positive for respiratory syncytial virus, irrespective of age, vis-à-vis percentage of ED visits due to bronchiolitis among children aged < 2 years.

### Additional viral characterisation

#### 2021–22

Of the 4,636 samples positive for influenza virus from all hospitalised patients between week 40 2021 and week 15 2022, 4,609 (99.4%) were type A, 20 (0.4%) were type B and 1 (0.02%) sample had both type A and type B; typing was not reported for 6 (0.13%) samples. Subtyping results of influenza A virus were available from one hospital laboratory: all 103 influenza A viruses subtyped were A(H3N2). All the 299 samples positive for influenza virus from primary care sentinel clinics between week 40 2021 and week 14 2022 were influenza A(H3N2).

#### 2022–23

Of the 7,348 samples from hospitalised patients between week 40 2022 and week 15 2023 positive for influenza virus, 5,739 (78.1%) were type A, 1,603 (21.8%) were type B and 6 (0.08%) samples had both type A and type B. Only two hospital laboratories subtyped influenza A viruses during the 2022–23 season. All 854 type A influenza viruses detected in those two hospitals underwent subtyping: 649 (76.0%) of them were A(H1N1)pdm09 and 156 (18.3%) were A(H3N2). Subtyping was unsuccessful for 49 (5.7%) samples which had Cq ≥ 36.

Of the 328 samples positive for influenza virus from primary care sentinel clinics between week 40 2022 and week 15 2023, 247 (75.3%) were influenza A and 81 (24.7%) were influenza B. Of the 247 influenza type A viruses, 198 (80.2%) were A(H1N1)pdm09 and 44 (17.8%) were A(H3N2). Subtyping was unsuccessful for 5 (2.0%) samples which had Cq ≥ 36.

### Analysis of respiratory syncytial virus out-of-season outbreak of 2021

We analysed data of RSV-positive patients admitted between week 19 and 30 2021, starting in week 22 which covered the first three weeks (weeks 19–21) of the outbreak.

**Figure 4:**
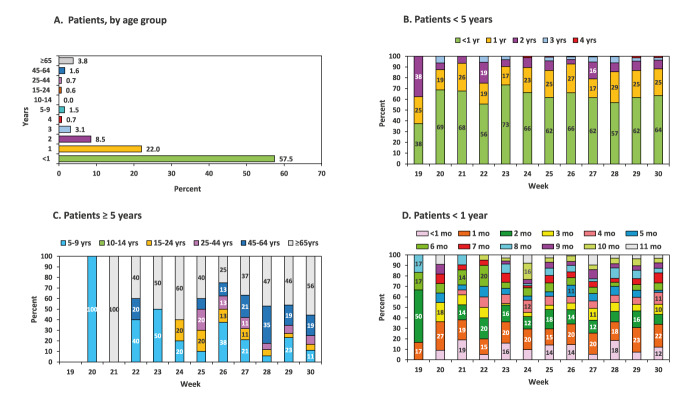
Percentage of patient samples tested positive for respiratory syncytial virus, by age group, Israel, 2021 (n = 1,608)

A total of 1,886 RSV-positive hospitalised patients were identified between week 19 and 30 2021. A total of 1,608 of them were identified in the national population registry and analysed by age group (Figure 4A). Specifically, 924 (57.5%) of these patients were aged < 1 years, 354 (22.0%) were in their second year of life (marked as 1 y), 137 (8.5%) were in their third year of life (marked as 2 y), 50 (3.1%) were in their fourth year of life (marked as 3 y) and 61 (3.8%) were aged ≥ 65 years. Each of the other age groups included ≤ 1.5% of the patients. Figure 4B demonstrates the weekly distribution of patients aged < 5 years, while Figure 4C demonstrates the weekly distribution of patients aged ≥ 5 years, by age group. Of the 131 patients who were aged ≥ 5 years, the largest age group of RSV-positive patients was ≥ 65 years old, consisting of 61 (46.6%) patients.

mo: month; yr: year.

Panel A (n =1,608); Panel B (n =1,477); Panel C (n = 131); Panel D (n = 924).

In panels B, C and D percentages are demonstrated only if they surpass 10%.

Of the 924 RSV-positive patients aged < 1 year, the highest number of patients were in their first 3 months of life: 109 (11.8%) were aged < 1 month, 186 (20.1%) were in their second month of life and 123 (13.3%) in their third month of life (Figure 4D).

**Figure 5:**
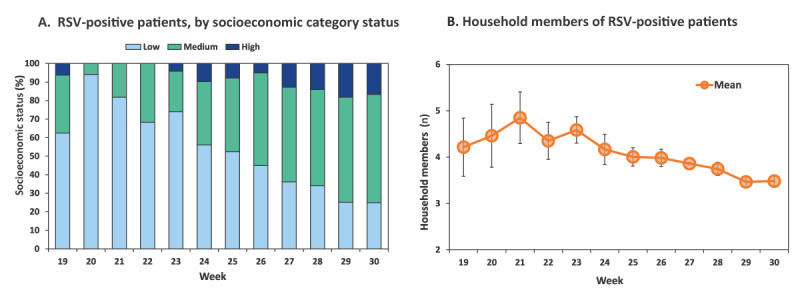
Socioeconomic status category (A) and mean number of household members (B) of hospitalised patients tested positive for respiratory syncytial virus, by week, Israel, week 19–30 2021

Of the 1,608 patients that were identified in the national population registry, SGA was identified for 1,547 patients, allowing the determination of the associated SES cluster and HHM. The initial weeks of the RSV outbreak were characterised by the predominance of hospitalised patients from a low SES category (Figure 5A). However, as the outbreak progressed, the percentage of patients from the low SES category decreased and the percentage of patients from higher SES categories increased (Figure 5A). Independent samples Kruskal-Wallis test demonstrated that the differences in SES categories among the weeks were statistically significant (p < 0.01). Multiple pairwise comparisons demonstrated statistically significant differences (p value < 0.01) between SES categories of each of weeks 20 to 26 and each of the weeks 29 to 30, between each of the weeks 20 to 23 and each of the weeks 27 to 28 and between weeks 23 and 26. There was also a statistically significant difference between SES categories of hospitalised patients with RSV in weeks 20–21 and week 26, as well as between week 25 and 28 (p < 0.05), as can be seen in Supplementary Table S1.

RSV: respiratory syncytial virus.

In panel B, the centre of each circle represents the mean and the error bars indicate 95% confidence intervals.

Between week 19 and 25, the mean number of HHM of RSV patients was 4–4.8, while between week 28 and 30 it was 3.5–3.7 (Figure 5B). The differences in the number of HHM in these weeks were statistically significant (p < 0.01). Multiple pairwise comparisons demonstrated statistically significant differences between the HHM numbers in weeks 21–26 and in weeks 29–30 (p < 0.01), between weeks 25–28 and weeks 21 and 23 (p < 0.01), between week 27 and 29 (p < 0.01) and between additional early and later weeks of the evaluation period (p < 0.05), as shown in Table S2.

Next generation sequencing of RSV genome was performed and compared to RSV-A and RSV-B reference strains. Of the 53 samples successfully sequenced, we identified 9 RSV-A and 44 RSV-B. As only a few RSV-A cases were identified, we continued analyses for RSV-B only, using sequences with > 80% coverage of the reference RSV-B genome (AY353550) (n = 37). The genomes of RSV-B were compared to whole genome reference strains, shown in Supplementary Figure S1A. A phylogenetic tree was constructed for the G protein only. The phylogenetic analysis showed that all Israeli RSV-B sequenced viruses were genetically closest to the reference RSVB/Argentina/BA-1607–04/DQ227397/2004/BA3 genotype, shown in Supplementary Figure S1A. In addition, the outbreak RSV B genotypes were not clustered by the hospital from which they originated, as presented in Supplementary Figure S1B.

### Contribution of the new surveillance platform to domains/objectives of the mosaic framework

Table shows an analysis of the contribution of the hospital laboratory surveillance platform to the domains and objectives of the mosaic framework. Based on our evaluation, the new surveillance platform contributed to objectives of all three domains. Specifically, data gathered by the new platform could contribute to two of the three objectives of Domain I, three of the four objectives of Domain II and one of the seven objectives of Domain III (Table). It can potentially contribute to one additional objective of Domain I and three additional objectives of Domain III, by obtaining additional information regarding the patients identified by the platform and/or by performing additional analyses of their samples.

**Table:** Contribution of a new hospital laboratory surveillance to the mosaic framework of surveillance for respiratory viruses of epidemic and pandemic potential, Israel

Domain	Domain description	Objectives	Likely contribution of the new surveillance platform to the mosaic platform domains and objectives	Contribution details
I	Detection and assessment of an emerging or re-emerging respiratory virus	1. Rapidly detect emerging or re-emerging respiratory virus outbreaks and other events	Yes	Out-of-season RSV outbreak detected
		2. Assess transmissibility, risk factors for transmission and extent of infection from an emerging or re-emerging respiratory virus	Yes	Extent of infection with influenza virus /RSV among newly admitted hospitalised persons
		3. Describe clinical presentation and risk factors for severe outcomes associated with an emerging or re-emerging respiratory virus	Potentially	Information can be obtained based on data collected
II	Monitor epidemiological characteristics of respiratory viruses in interpandemic periods	1. Monitor epidemiologic and clinical characteristics of illness over time	Yes	Epidemiological characteristics for out-of-season RSV outbreak Clinical characteristics can be obtained based on data collected
		2. Monitor virologic and genetic characteristics of circulating viruses	Yes	Whole genome sequencing of outbreak viruses
		3. Monitor situation in high-risk settings and vulnerable populations	Yes	Monitor RSV/influenza virus activity in hospitalised patients by age group.
		4. Monitor impact on and coping abilities of healthcare systems	No	Not applicable
III	Informing use of human health interventions	1. Monitor the impact of non-medical interventions in the population	Yes	Monitor RSV/influenza virus activity in hospitalised patients before and after intervention
		2. Provide candidate vaccine viruses for vaccine composition, production, and risk assessment	Potentially	Samples can be requested from hospital laboratories
		3. Monitor vaccine coverage, effectiveness, impact and cost-effectiveness	Potentially	The data collected can be used for vaccine effectiveness estimation and vaccine impact. (It is not expected to contribute to vaccine coverage or cost-effectiveness assessments)
		4. Monitor the effectiveness of antivirals and other therapeutics	Potentially	Requires additional data from medical records
		5. Monitor the effectiveness of diagnostic tests	No	Not applicable
		6. Monitor the effectiveness of clinical care pathways, including Infection, Prevention and Control (IPC)	No	
		7. Monitor adverse events to vaccines and therapeutics	No	

RSV: respiratory syncytial virus.

## Discussion

The COVID-19 pandemic challenged respiratory surveillance systems globally [3] with health authorities around the world adopting various strategies to monitor the progress of the pandemic [12]. However, as the world is moving away from the pandemic stage of COVID-19, some of the adopted strategies require re-evaluation. Specifically, there is a need to consider which strategies should remain active during the post-pandemic period, while preparing for potential future global outbreaks. The mosaic surveillance framework developed by the WHO was designed to assist countries with identifying monitoring goals for respiratory viruses and implementing methods to achieve them [1]. The mosaic framework focuses primarily on enhancing surveillance between pandemics which will improve the prospects of early warning [11].

The purpose of establishing the hospital respiratory virus surveillance system in Israel was to support the sentinel surveillance system with the identification of the activity of influenza virus and RSV. Based on previous experience which showed that RSV activity pattern changed after the appearance of the pandemic influenza A(H1N1)pdm09 [13], we were concerned that the activity patterns of influenza virus and RSV may change during the COVID-19 pandemic as well, and that the primary care sentinel surveillance system would not suffice to characterise such changes, especially if viral circulation is low.

Our evaluation demonstrated that the hospital respiratory virus laboratory surveillance system, since becoming operational, had a significant role in providing a reliable pattern of the activity of influenza virus and RSV.

During the evaluation period, which spanned three seasons, the new system captured the lack of seasonal influenza activity during the 2020–21 autumn-winter season, the shorter than usual 2021–22 season and the comeback influenza activity of the 2022–23 season. These findings were consistent with the primary care sentinel surveillance findings.

The new system also captured RSV activity during the same period. Specifically, the low and sporadic activity during the 2020–21 season, the out-of-season 2021 outbreak which merged with a delayed 2021–22 autumn-winter RSV activity and the comeback RSV activity of the 2022–23 season.

Only a few surveillance systems around the world have had a comprehensive hospital-based surveillance of respiratory viruses, which is not based on the SARI case definition. The Health and Human Services (HHS)-Protect hospitalisation surveillance system was established in the US in March 2020 to facilitate public health response to the COVID-19 pandemic [14]. All hospitals in the US were required to report laboratory-confirmed COVID-19 and influenza hospitalisations [15]. The reported number of new influenza hospitalisations were presented in the weekly Influenza Surveillance Report [16]. However, since the HHS-Protect and the subsequent hospital reporting platform, the Centers for Disease Control and Prevention (CDC) National Healthcare Safety Network (NHSN), which operated between December 2022 and April 2024, did not collect data on the number of patients tested for the influenza virus [17,18], no test-positivity was calculated [15]. Furthermore, the HHS-Protect did not include RSV reporting and the NHSN RSV reporting was only optional [17]. In Queensland, Australia, hospitalisations of laboratory-confirmed influenza and RSV patients in public hospitals are also reported [19] without calculation of the test-positivity rate.

The hospital surveillance platform established in Israel has several advantages. Firstly, it relies on hospitals’ existing resources without any external intervention with sampling decisions i.e. samples are taken based on the medical team clinical decision or hospital criteria, where existing. In comparison, the SARI surveillance system, which operated in Israel before the COVID-19 pandemic, required multiple resources in terms of work force, testing kits and cooperation of medical teams. During a pandemic, when health systems are under pressure due to excessive workload, it would be impractical to direct resources towards a case-definition-based hospital surveillance system. Secondly, the data from 26 hospitals constitute a considerable sample size, which surpasses those of the primary care sentinel and the SARI surveillance platforms in Israel. Thirdly, this system generates data continuously throughout the year, compared with our primary care sentinel clinics and SARI surveillance platforms, which usually operate during the autumn-winter months (except during the COVID-19 pandemic, when primary care sentinel clinics operated also during other periods of the year). The large sample size and the continuous data availability allowed for the detection of the out-of-season RSV outbreak at a time when there was no sentinel surveillance in primary care. Also, the early increase in influenza activity several weeks before week 40 2022 could be caught, ahead of the primary-care sentinel clinics activity period.

The hospital surveillance system fulfils several objectives in each of the three mosaic framework domains. For example, the detection of viral activity is consistent with Objective 1 of Domain I [11] and assessing the burden of hospitalisations associated with each virus is consistent with Objective 2 of Domain I.

Furthermore, the information collected by this surveillance platform helped conduct epidemiological investigations, consistent with Objective 1 of Domain II [11]. Samples collected by hospitals participating in this surveillance approach were used for virological and genomic analyses, consistent with Objective 2 of Domain II [11].

Since each positive sample reported by hospitals was accompanied by information on age, sex and admission department of the patient, morbidity level of each season could be assessed [20]. The data also enabled us to determine the number and percentage of deaths among laboratory-confirmed patients and stratify them by age group [20]. Furthermore, geostatistical data could potentially assist in assessing the presence or absence of space-time clusters, their geographic location [21] and the characteristics of the population affected. In this regard, by using geostatistical data of RSV-positive patients at the start of the out-of-season RSV outbreak, we were able to characterise the population affected by SES and HHM. Identifying population characteristics by location, age and social parameters can assist in the implementation of public health measures. Moreover, the potential to nationally monitor the activity of other respiratory viruses using this hospital surveillance platform, exists.

The outbreak data collected by the hospital surveillance platform helped mobilise an ad hoc intervention against RSV. During the 2021 out-of-season RSV outbreak, the only health intervention available was palivizumab, a monoclonal antibody serving as passive vaccine approved for use in at-risk infants [22]. However, palivizumab has been approved for use in Israel only between the months November and March. Following the presentation of the outbreak analysis by the ICDC to the Israel Vaccine Advisory Committee, an ad hoc recommendation was made by the committee calling the Israel Ministry of Health to support and budget the administration of palivizumab out-of-season [23].

The Israel hospital surveillance platform has several disadvantages. Firstly, patient sampling is not performed based on a specific case definition. Thus, sampling frequency and approach may vary between hospitals and medical teams. We did not gather information on decisions for hospital admission or clinical data in real-time. However, we demonstrated the consistency of this surveillance platform with other surveillance platforms, the ability to perform outbreak investigation and mobilise a preventive health intervention based on the data gathered.

## Conclusion

The hospital laboratory surveillance platform has been an important addition to the Israeli respiratory virus surveillance during and following the pandemic. It contributed to the WHO mosaic framework without requiring multiple resources. The potential contribution of this surveillance platform to several other mosaic platform objectives requires additional data and further evaluation.

## Supplementary Data

554000 Supplement
