# Supplementary material for: Hospital surveillance of respiratory viruses during the COVID-19 pandemic and beyond: contribution to the WHO mosaic framework, Israel, 2020 to 2023
Source: Euro Surveill. 2024 Aug 8;29(32):2300634. doi: 10.2807/1560-7917.ES.2024.29.32.2300634 (PMC11312018; doi:10.2807/1560-7917.ES.2024.29.32.2300634)

Supplementary materials

"This supplementary material is hosted by *Eurosurveillance* as supporting information alongside the article '**Hospital surveillance of respiratory viruses during the COVID-19 pandemic and beyond: contribution to the WHO mosaic framework, Israel, 2020 to 2023'**, on behalf of the authors, who remain responsible for the accuracy and appropriateness of the content. The same standards for ethics, copyright, attributions and permissions as for the article apply. Supplements are not edited by *Eurosurveillance* and the journal is not responsible for the maintenance of any links or email addresses provided therein."

Contents Page

Table S1. Multiple pairwise comparisons of SES categories, RSV off-season outbreak, weeks 19 to 30, 2021, Israel....................2

Table S2. Multiple pairwise comparisons of number of household members, RSV off-season outbreak, weeks 19 to 30, 2021, Israel....................2

Figure S1. Phylogenetic analysis of RSV B sequences from samples obtained at different hospitals in Israel during the 2021 off-season outbreak

- **(A)** Phylogenetic tree representing G protein genes of 37 samples along with 26 RSV references....................3
- **(B)** Phylogenetic tree representing a closer look at the RSV from samples obtained at from different hospitals in Israel....................4

Table S1. Multiple pairwise comparisons of SES categories among RSV-positive patients, off-season outbreak, weeks 19 to 30, 2021, Israel

	Week 19	Week 20	Week 21	Week 22	Week 23	Week 24	Week 25	Week 26	Week 27	Week 28	Week 29	Week 30
**Week 19**		1.000	1.000	1.000	1.000	1.000	1.000	1.000	1.000	1.000	0.192	0.226
**Week 20**	1.000		1.000	1.000	1.000	0.321	0.144	0.028	0.001	0.000	0.000	0.000
**Week 21**	1.000	1.000		1.000	1.000	0.551	0.182	0.019	0.000	0.000	0.000	0.000
**Week 22**	1.000	1.000	1.000		1.000	1.000	1.000	0.516	0.004	0.001	0.000	0.000
**Week 23**	1.000	1.000	1.000	1.000		1.000	0.182	0.004	0.000	0.000	0.000	0.000
**Week 24**	1.000	0.321	0.551	1.000	1.000		1.000	1.000	0.305	0.066	0.000	0.000
**Week 25**	1.000	0.144	0.182	1.000	0.182	1.000		1.000	0.246	0.037	0.000	0.000
**Week 26**	1.000	0.028	0.019	0.516	0.004	1.000	1.000		1.000	0.356	0.000	0.000
**Week 27**	1.000	0.001	0.000	0.004	0.000	0.305	0.246	1.000		1.000	0.478	0.745
**Week 28**	1.000	0.000	0.000	0.001	0.000	0.066	0.037	0.356	1.000		1.000	1.000
**Week 29**	0.192	0.000	0.000	0.000	0.000	0.000	0.000	0.000	0.478	1.000		1.000
**Week 30**	0.226	0.000	0.000	0.000	0.000	0.000	0.000	0.000	0.745	1.000	1.000	

Table S2. Multiple pairwise comparisons of number of household members among RSV-positive patients, off-season outbreak, weeks 19 to 30, 2021, Israel

	Week 19	Week 20	Week 21	Week 22	Week 23	Week 24	Week 25	Week 26	Week 27	Week 28	Week 29	Week 30
**Week 19**		1.000	0.786	1.000	0.987	1.000	1.000	1.000	0.988	0.902	0.281	0.317
**Week 20**	1.000		0.992	1.000	1.000	0.998	0.917	0.880	0.608	0.314	0.020	0.025
**Week 21**	0.786	0.992		0.759	0.992	0.125	0.007	0.003	0.000	0.000	0.000	0.000
**Week 22**	1.000	1.000	0.759		0.994	0.999	0.860	0.770	0.309	0.064	0.000	0.000
**Week 23**	0.987	1.000	0.992	0.994		0.352	0.007	0.001	0.000	0.000	0.000	0.000
**Week 24**	1.000	0.998	0.125	0.999	0.352		0.997	0.987	0.630	0.127	0.000	0.000
**Week 25**	1.000	0.917	0.007	0.860	0.007	0.997		1.000	0.993	0.625	0.000	0.001
**Week 26**	1.000	0.880	0.003	0.770	0.001	0.987	1.000		0.996	0.589	0.000	0.000
**Week 27**	0.988	0.608	0.000	0.309	0.000	0.630	0.993	0.996		0.996	0.007	0.014
**Week 28**	0.902	0.314	0.000	0.064	0.000	0.127	0.625	0.589	0.996		0.159	0.247
**Week 29**	0.281	0.020	0.000	0.000	0.000	0.000	0.000	0.000	0.007	0.159		1.000
**Week 30**	0.317	0.025	0.000	0.000	0.000	0.000	0.001	0.000	0.014	0.247	1.000	

P-value <0.01 in orange cells; p-value <0.05 in green cells

Figure S1. Phylogenetic analysis of RSV B sequences from samples obtained at different hospitals in Israel during the 2021 off-season outbreak.

(A) Phylogenetic tree representing G protein genes of 37 samples along with 26 RSV references.

A

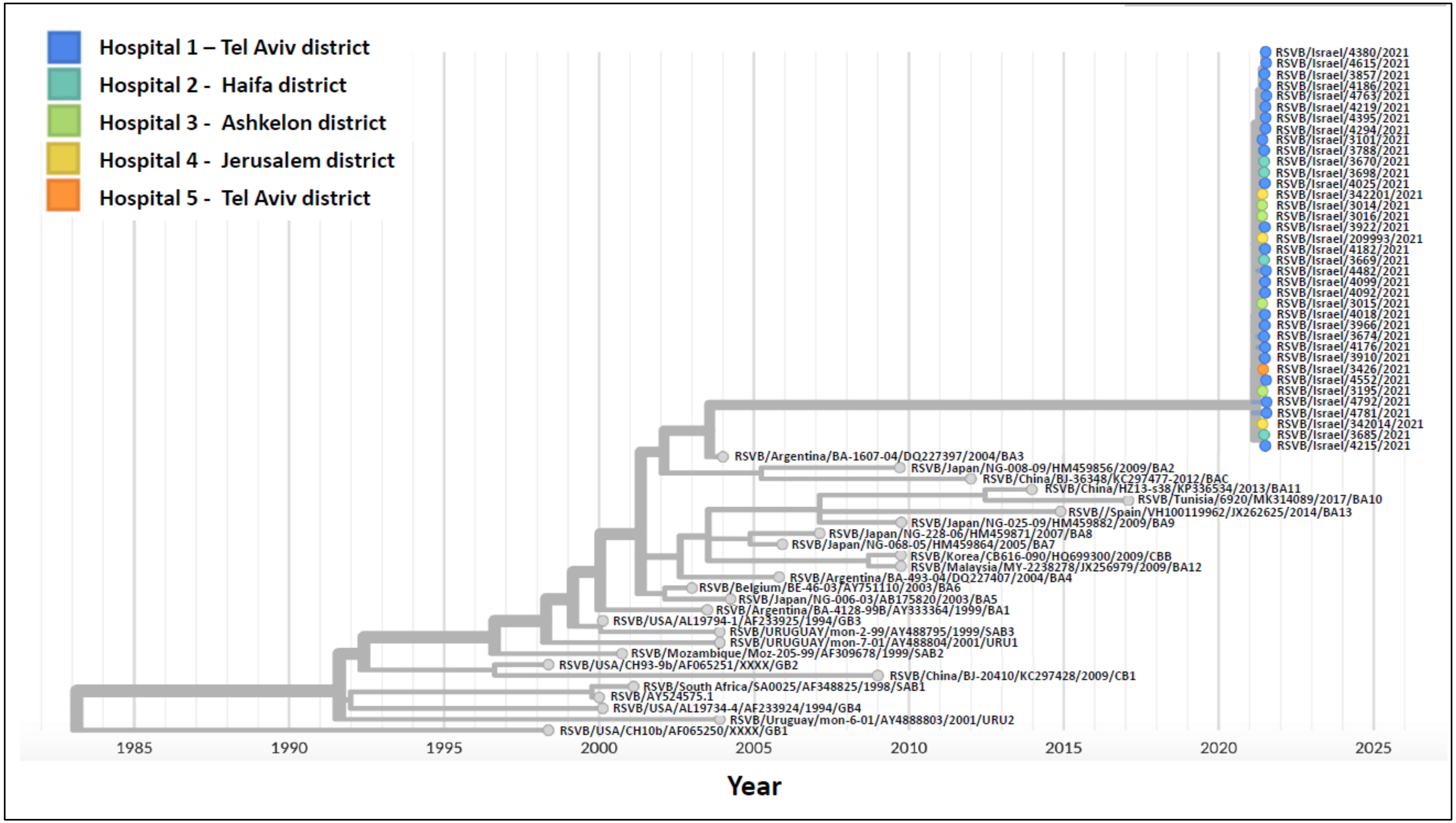

(B) Phylogenetic tree representing a closer look at the RSV using whole genome sequences from samples obtained at different hospitals in Israel.

B

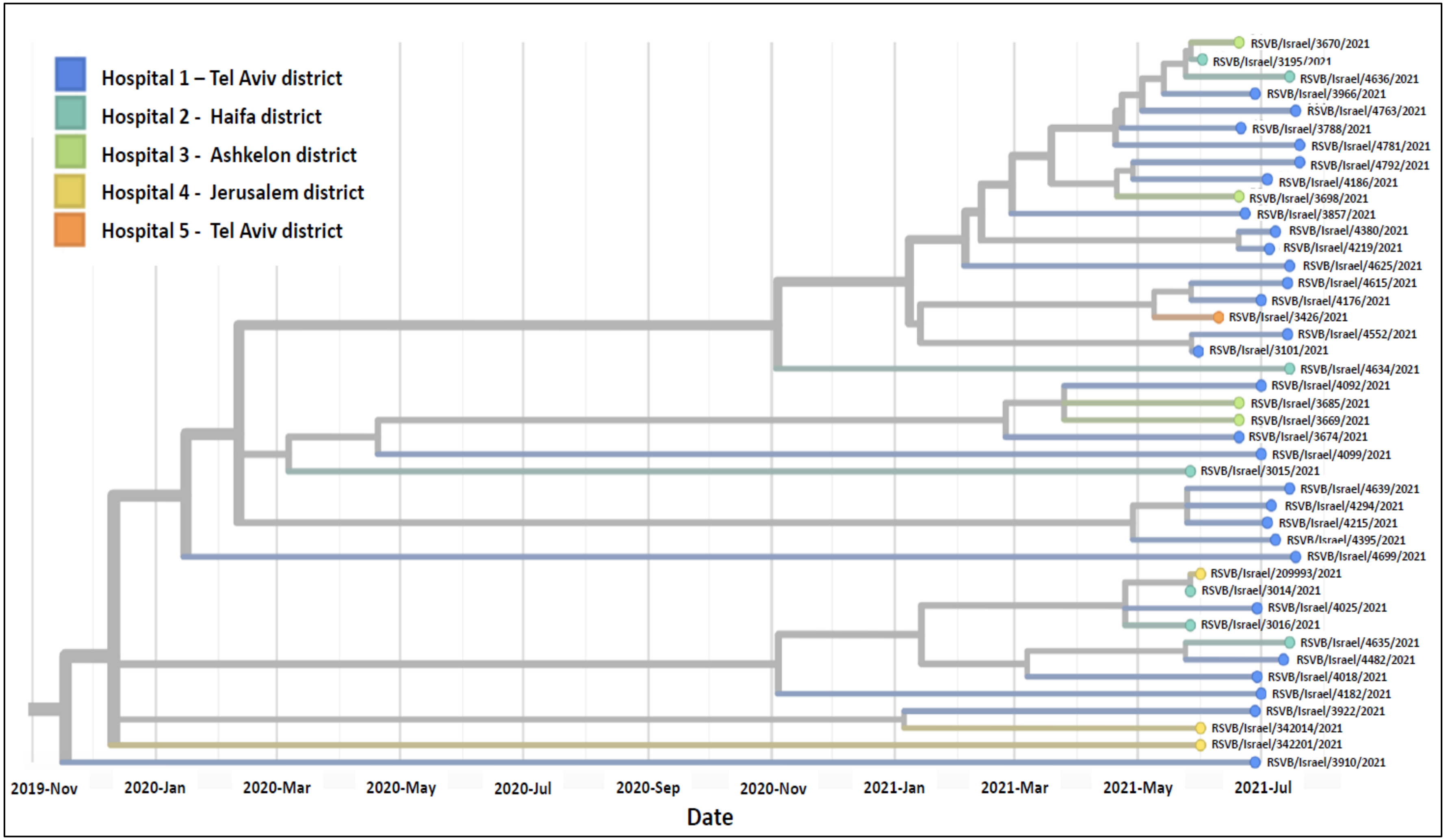

Supplement: Supplement [file 23-00634_GLATMAN-FREEDMAN_Supplement.pdf]
